# Expression of EpCAM Increases in the Hepatitis B Related and the Treatment-Resistant Hepatocellular Carcinoma

**DOI:** 10.1155/2014/172913

**Published:** 2014-02-17

**Authors:** Osamu Kimura, Yasuteru Kondo, Takayuki Kogure, Eiji Kakazu, Masashi Ninomiya, Tomoaki Iwata, Tatsuki Morosawa, Tooru Shimosegawa

**Affiliations:** Division of Gastroenterology, Tohoku University Hospital, 1-1 Seiryo-Machi, Aoba-ku, Sendai, Miyagi 980-8574, Japan

## Abstract

Increasing evidence supports the important role of cancer stem cells (CSCs). Many reports suggest that epithelial cell adhesion molecule (EpCAM) is a useful marker for cancer stem cells in hepatocellular carcinoma (HCC). To elucidate the mechanisms of cancer stem cells, the development of specific molecular targeted drugs has become very important. In the present study, we examined the EpCAM expression pattern and its characteristic expression in resected HCC. We studied the drug resistance of EpCAM expression cells. EpCAM expression was detected significantly more frequently with hepatitis B virus (HBV) than with other etiologies. In HCC resection patients who had received prior treatment (transcatheter arterial embolization or hepatic arterial infusion chemotherapy), EpCAM was strongly expressed. In particular, very strong expression was observed after hepatic arterial infusion chemotherapy. The PLC/PRF/5 human HCC cell line expressed bimodal EpCAM, and EpCAM-positive cells had CSC cell potency. The EpCAM expression in EpCAM-positive cells increased significantly by treatment with cisplatin. EpCAM-positive cells showed better viability than EpCAM-negative cells when treated with ciplatin. Collectively, our results suggest that cancer stem cells are highly expressed in hepatitis B and have potential anticancer drug resistance.

## 1. Introduction

Worldwide, hepatocellular carcinoma (HCC) is the fifth most frequently diagnosed cancer [1] in human; however, it is the second most frequent cause of cancer death. In the early stages of hepatocellular carcinoma, when patients still maintain a hepatic functional reserve, local treatment such as hepatic resection or radiofrequency ablation is relatively effective [2]; however, many patients have repeated recurrence and died. For advanced hepatocellular carcinoma, hepatic arterial infusion chemotherapy (HAIC) is sometimes effective [3, 4]. Also, recently sorafenib [5] has come into use, but satisfactory results [6] have not been shown yet. Therefore, considering the current therapy options for HCC, finding a new therapeutic target molecule has become very important.

Recently, there have been many studies [7–9] about cancer stem cells. Among heterogenous cell populations, the role of a relatively small fraction with potent growth potential, so-called cancer stem cells (CSCs), in tumorigenicity has been emerging. CSCs are estimated to comprise about 0.03–29% of tumor cells [10]. If CSCs survive treatment with anticancer drugs, a small number of cancer stem cells can grow again, acquiring the ability to resist anticancer drugs. Therefore, the development of a treatment with a molecular mechanism for cancer stem cells is important.

Some markers of CSCs in HCC have been reported (i.e., CD133 [11, 12], CD90 [13], CD13 [14], and epithelial cell adhesion molecule (EpCAM) [15–17]). EpCAM is intricately linked with the cadherin-catenin pathway, and the fundamental Wnt/*β*-catenin signaling pathway is responsible for intracellular signaling and polarity [18]. At first, EpCAM was reported as a marker of the cancer stem cell in the pancreatic carcinoma and the breast cancer. In addition, EpCAM appears in human hepatic progenitor cell. Therefore, we thought that EpCAM could become a useful cancer stem cell marker in a HCC. We have reviewed EpCAM in the previous reports. We have reported that PLC/PRF/5 expressed bimodal EpCAM [17]. The EpCAM-positive cell subpopulation showed higher colony formation and higher expression of other putative CSC markers (i.e., CD133, CD90, and ABCG2) than did EpCAM-negative cells. Furthermore, the bifurcate differentiation from EpCAM-positive cells into both EpCAM-positive and EpCAM-negative cells was obvious both *in vitro* and *in vivo*, while EpCAM-negative cells sustained their phenotype. Sorted EpCAM-positive and EpCAM-negative populations from PLC/PRF/5 were separately inoculated in NOD/scid/*γ*  c^null^ (NOG) mice, and the growth was monitored. EpCAM-positive cells needed a lower number to form a tumor than did EpCAM-negative cells. In addition, EpCAM-positive cells showed earlier onset and formed larger tumors than did the same number of EpCAM-negative cells. Accordingly, we considered that EpCAM-positive PLC/PRF/5 cells could be used as a model CSC cell line.

In the present study, we examined the EpCAM expression pattern and characteristics of EpCAM expression in HCC using resected HCC specimens. Additionally, we conducted *in vitro* studies of EpCAM-expressing PLC/PRF/5 cells.

## 2. Materials and Methods

### 2.1. Patients

Specimens were obtained during operations for HCC lesions from 2005 to 2010 in Tohoku University Hospital (*n* = 71). 58 patients had surgery without previous treatment. The other 13 patients received previous treatment (transcatheter arterial chemoembolization (TACE), *n* = 9; hepatic arterial infusion chemotherapy (HAIC), *n* = 4) before the operation.  Table 1 shows the clinical profiles of the 58 HCC patients. In our hospital, the HAIC protocol was fluorouracil (5-FU) (250 mg/day for 5 days/week for 4 weeks), cis-diamminedichloroplatinum (CDDP) (10 mg–5 mg/day for 5 days/week for 4 weeks), and levofolinate calcium 100 mg. These drugs were administered continuously using an ambulatory balloon infusion pump. Most of the patients received 2 courses of the treatment to shrink the tumor prior to surgery.

Written informed consent was obtained from each patient, and the study was approved by the Ethics Committee of Tohoku University School of Medicine (number 2008-241). We analyzed the correlation between EpCAM expression and the patient's characteristics such as etiology, age, gender, tumor size, liver status, and tumor factors.

### 2.2. Immunohistochemical Staining

Formalin-fixed and paraffin-embedded tissues were sectioned into 2 mm-thick slices. They were heated twice for 5 minutes in 10 mM citrate buffer (pH 6.1, Target Retrieval Solution; Dako, Glostrup, Denmark) in a microwave oven after deparaffinization. The specimens were treated with 3% hydrogen peroxide in methanol for 10 minutes and incubated with an antiepithelial specific antigen (ESA) antibody (Ab) [B29.1 VU-ID9] (Gene Tex, Irvine, CA) at 4°C overnight. After incubation with peroxidase-conjugated anti-mouse IgG Ab as a secondary Ab (Nichirei, Tokyo, Japan) for 30 minutes at room temperature, the sections were developed using a NovaRED substrate kit (Vector laboratories, Burlingame, CA) and counterstained with hematoxylin.

### 2.3. Cell Lines and Cell Cultures

Human HCC cell lines Huh7, HepG2, Hep3B, Li-7, and PLC/PRF/5 were obtained from the Cell Resource Center for Biomedical Research, Tohoku University. Cells were cultured in DMEM (high glucose) with L-glutamine and phenol red (Wako, Osaka, Japan) supplemented with 10% heat-inactivated fetal bovine serum (FBS) (Sigma-Aldrich) at 37°C in 5% CO_2_ atmosphere.

### 2.4. Antibodies and Flow Cytometry Analysis

The following monoclonal antibodies were used. The biotinylated-anti-ESA (EpCAM) Ab was from Gene Tex. Streptavidin-PE (BD biosciences, San Jose, CA) was used as a secondary reagent for biotinylated Abs. The HCC cell lines were detached with Accutase (Chemicon, Billerica, CA) for 15 minutes at 37°C. The dissociated cells were washed twice with phosphate buffered saline (PBS) and resuspended with staining buffer (PBS containing 0.5% bovine serum albumin (BSA), 2 mM ethylenediaminetetraacetic acid (EDTA), and 20 mM HEPES) at 1 × 10^6^/100 *μ*L. FcR-blocking reagent (Miltenyi Biotec) was added to inhibit the unspecific binding of antibodies. Cells were stained for 40 minutes on ice. Dead cells were eliminated using propidium iodide (PI). Flow-cytometric analysis was performed by FACS Canto II (BD biosciences), and the collected data were analyzed using FACS Diva software (BD biosciences). Gating was set based on the isotype-staining profiles.

### 2.5. Cell Sorting

Cell lines were stained using the same protocol as for the flow-cytometric analysis. Cell sorting was performed by FACS Aria II (BD biosciences) using FACS Aria's purity sorting mode. The purity of the sorted cells was evaluated by flow cytometry. The purity after sorting was typically more than 95%.

### 2.6. Statistical Analysis

Statistical analyses were performed using software JMP.  Table 1 was evaluated with Kruskal-Wallis test or *X* ^2^ test. Figures 2 and 4 were evaluated with Fisher's exact test. Figures 3 and 5 were evaluated with Student's *t*-test. Values of *p* < 0.05 were considered statistically significant.

## 3. Results

### 3.1. EpCAM Expression in Nontumor Tissues and Tumor Tissues

**Figure 1:**
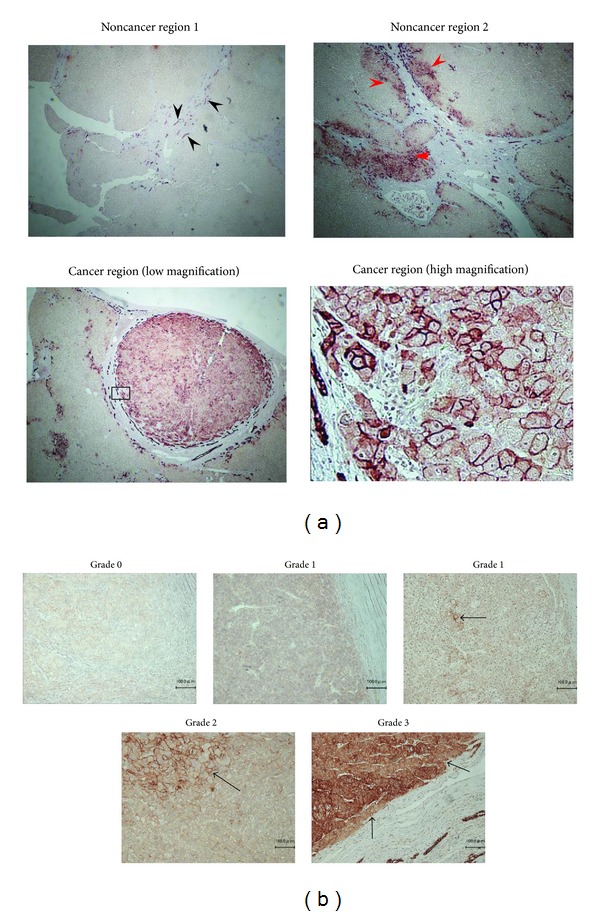
Immunohistochemical staining of EpCAM in resected HCCs. (a) EpCAM expression was observed in bile duct (black arrows). Many hepatocytes did not express EpCAM, but it was expressed in the regenerated damaged liver tissue like that caused by cirrhosis (red arrows). EpCAM expression in hepatocellular carcinoma (under panels). (b) Black arrows indicate cells with high EpCAM expression in HCC. EpCAM (Grade 0 (0%), Grade 1 (<10%, or diffusely weakly expression), Grade 2 (≥10% and <50%, resp.), and Grade 3 (≥50%)).

Significant difference in the clinical profiles, except age and ALT, was not accepted (Table 1), because cancer occurs in hepatitis B virus (HBV) patients earlier than in those with other etiologies, and [non-B, non-C (NBNC) hepatitis] patients had relatively normal livers. In nontumor tissues, cholangiocytes and normal hepatocytes expressed EpCAM. In particular, EpCAM was expressed at the regenerating region in normal hepatocytes (Figure 1(a)). On the other hand, the stained area and intensity varied in the HCC tissues in each case. At high magnification, the EpCAM expression was heterogeneous. HCC tissues were divided into four grades according to the level of EpCAM (Grade 0 (0%), Grade 1 (<10% or diffusely weakly expression), Grade 2 (≥10% and <50%, resp.), and Grade 3 (≥50%)) (Figure 1(b)). Grades 1 to 3 were EpCAM positive, and Grade 0 was EpCAM negative.

### 3.2. High Expression of EpCAM in HBV Patients

**Figure 2:**
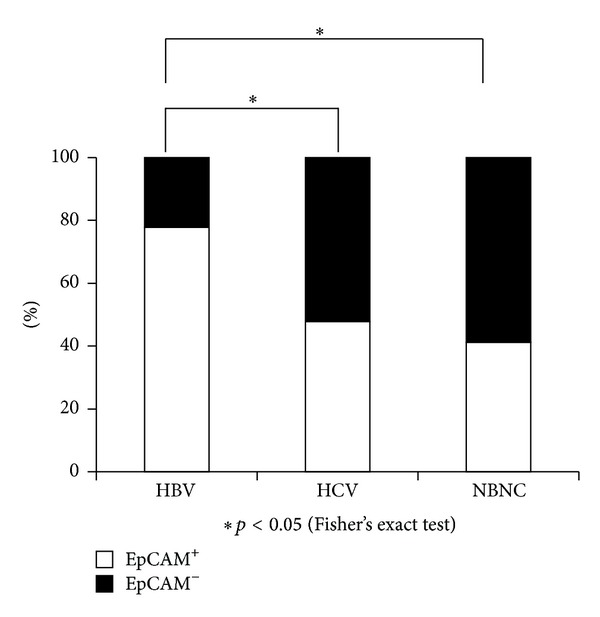
The frequency of EpCAM expression in each etiology. The frequency of EpCAM expression in resected HCCs was determined for EpCAM^+^ (white bars) and EpCAM^−^(black bars).

An analysis of 58 primary HCC tissues (etiology: HBV, *n* = 18; HCV, *n* = 23; NBNC, *n* = 17) was conducted.  Table 2 shows the EpCAM expression grades in the resected HCCs. No significant difference was recognized in each etiology according to the grade. Grade 0 was EpCAM negative, and Grades 1 to 3 were EpCAM positive. In 78% of the HBV patients (14/18), EpCAM expression was found in the resected HCC. Such expression was significantly higher than in those with other etiologies (HCV, 47%; NBNC, 41%) (Figure 2). No differences were found between HCV and NBNC patients.

**Figure 3:**
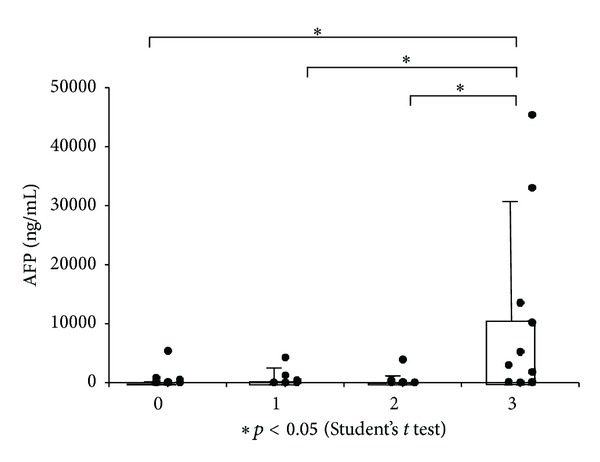
AFP of each grade of EpCAM expression. The average is shown with SD.

In Grade 3, a rise of the AFP was significant in comparison with the other grades (Figure 3); however, no other characteristics showed correlation with the EpCAM expression.

### 3.3. High Expression of EpCAM in Previously Treated HCC Resection

**Figure 4:**
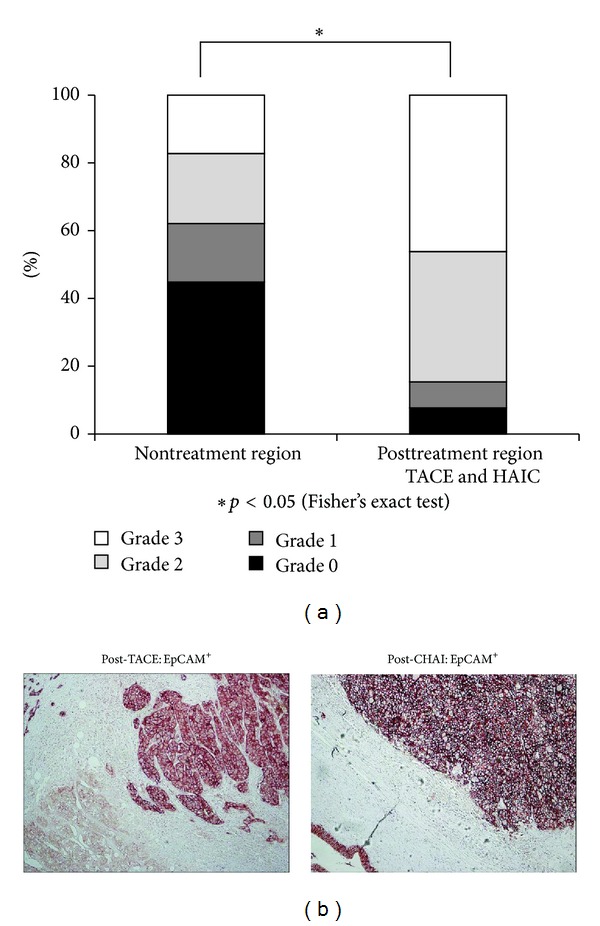
(a) Frequency of EpCAM expression in nontreated regions and posttreatment regions (TACE and HAIC). (b) Immunohistochemical staining of EpCAM in resected HCCs previously treated.

Either transarterial chemoembolization (TACE) or hepatic arterial infusion chemotherapy (HAIC) was performed on the patients prior to surgery. These patients were operated for residual HCC.  Table 3 shows the clinical profiles of the surgery alone patients (*n* = 58) and patients (*n* = 13) who received prior treatment (TACE or HAIC). Among the significant clinical profile differences, only the tumor size and Child-Pugh classifications were accepted. Those patients receiving the treatments before the resection showed very high expression of EpCAM (Figure 4(a)). In 92% of the patients that received previous treatment (12/13), EpCAM expression was found. In particular with patients who had resection after HAIC treatment, very strong expression was observed in all positive cases (Figure 4(b)).

### 3.4. EpCAM-Positive PLC/PRF/5 Cells Had Anticancer Drug Resistance Potency

**Figure 5:**
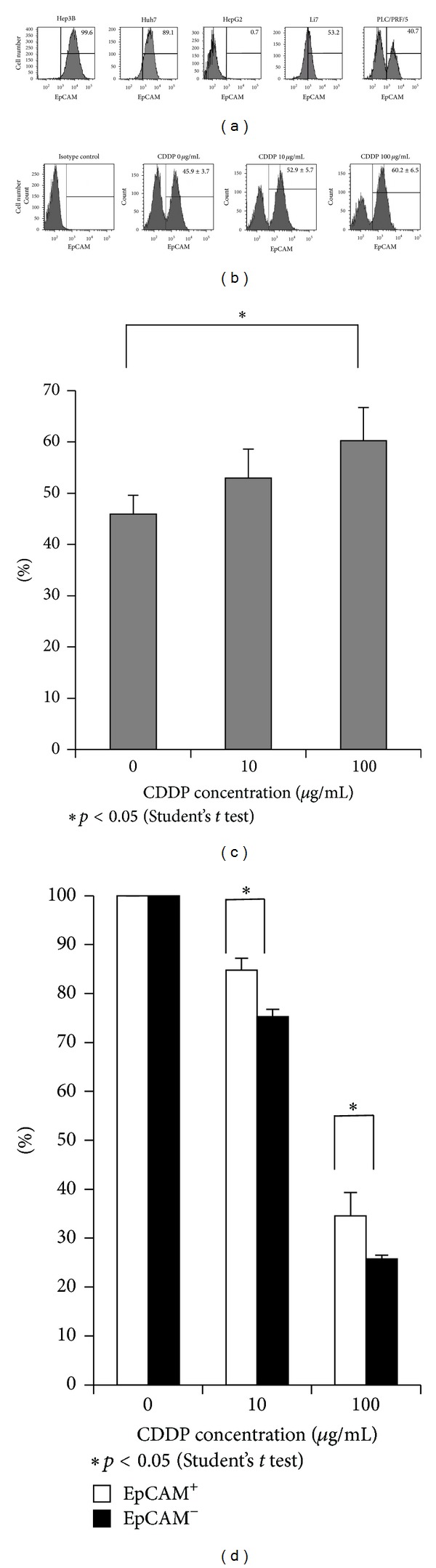
EpCAM-positive cells of PLC/PRF/5 had anticancer drug resistance potency. (a) Flow-cytometric analysis of various surface molecules in HCC cell lines. Percentages of the indicated molecule-positive cells are indicated. A representative result of three independent staining experiments is shown. (b)-(c) PLC/PRF/5 were exposed to various doses of cisplatin for 24 hr. The results of flow-cytometoric analysis are shown. The average of 3 independent experiments is shown with SD. (d) The cell viability was determined by MTS cell proliferation assay after exposure to various doses of cisplatin. The average of 3 independent experiments is shown with S.E.M.

The FACS analysis of 5 different HCC-derived cell lines showed various staining patterns (Figure 5(a)). Among them, PLC/PRF/5 showed a unique bimodal pattern of EpCAM expression. Previously, we reported [17] that EpCAM-positive cells had cancer stem cell (CSC) potency. In this study, we demonstrated that the expression of EpCAM had increased in the treatment-resistant HCC. We used PLC/PRF/5 to investigate the effect of anticancer drugs on the EpCAM-positive cells. PLC/PRF/5 cells showed increased frequency of EpCAM-positive cells depending on the concentration of cisplatin (Figures 5(b) and 5(c)). Next, we sorted PLC/PRF/5 [17] positive cells and negative cells using a cell sorter. The cells were exposed to various doses of cisplatin for 24 hrs. The cell viability was determined by MTS cell proliferation assay. EpCAM-positive cells showed better viability than EpCAM-negative cells after cisplatin treatment (Figure 5(d)).

## 4. Discussion

Many studies [15, 19] have reported that EpCAM-positive cells in HCC are cancer stem cells. In our previous study, we reported [17] that EpCAM-positive cells in PLC/PRF/5 had high tumorigenicity, high colony formation, and differentiation potency. We used *Lentivirus* to introduce EpCAM cDNA to EpCAM-negative cell clones; however, the CSC potency did not improve as compared to EpCAM-positive cells. Therefore, EpCAM-positive cells did not only have the EpCAM gene but also had the characteristics of cancer stem cells. For this reason, we think EpCAM serves well as a CSC marker in HCC.

Hepatitis B is known to develop HCC faster than other etiologies. Carcinogenesis from HBV appeared at ages about 10 years younger than that from other etiologies in this study. Although several mechanisms have been suggested to explain the formation of HCC in hepatitis B patients, the mechanism still remains uncertain [20]. In cancer stem cell theory, it is thought that hepatitis B patients are more susceptible to forming cancer stem cells than patients with other etiologies. Our present study also showed results supporting this presumption; however, the promoting factor for cancer stem cells in HBV was not revealed. Arzumanyan et al. [21] and Wang et al. [22] suggested that HBx promotes cancer stem cells with EpCAM by activating *β*-catenin and the epigenic upregulation of miR-181. In addition, Chisari and Ferrari reported that HBx protein generates cancer stem cells from hepatic progenitor cells. We think that the likelihood of HBx acting as a promoting factor for cancer stem cells is high but this would not explain everything, because HBs antigen causes cancer and forms a microenvironment that evades immunity [23]. Moreover, HBe antigen-transfected HepG2 cell lines showed the upregulation of EpCAM in our study (data not shown). Our present study did not suggest distinct differences between hepatitis C patients and non-C non-B hepatitis patients in terms of carcinogenesis. This is because, for hepatitis C, inflammation is a contributing factor for carcinogenesis as compared to hepatitis B. It has been reported that HCV core results in carcinogenesis at a high rate by continuously activating PPAR*α* [24]. We think that inflammation also induces the formation of cancer stem cells but that it is more complicated than HBV. Therefore, we think that it is very useful to clarify the mechanism of carcinogenesis resulting from HBV in order to explain the mechanisms for cancer stem cells. We think that many CSCs are included in the HBV-related HCC. Actually, high expression of EpCAM admits HBV-related HCC in other studies [25]. However, it cannot be said that the prognosis of the HCC from HBV is poor. Because overall survival of HCC is influenced from not only the extension of cancer but also the liver function. The hepatitis B patients who can control fibrosis of the liver with the antiviral drug are not inferior to other etiologies in overall survival.

HCV and NBNC patients did not express more EpCAM than HBV patients. It was shown that its effect on the cancer stem cells of HCV was lower than that on those of HBV. It is thought that, in HCV, CSCs are caused through oxidative stress.

Yamashita et al. described AFP^+^ EpCAM^+^ cells as a more precise marker of CSC [19]. Our results did not show any relation with AFP when the EpCAM expression level was low but, with Grade 3 expression, significant AFP expression was also shown. AFP is expressed in plasma so it cannot be simply compared, but we believe EpCAM serves well as a marker for locating the cancer stem cells.

Generally, it is thought that HCC has anticancer drug resistance. Even though the anticancer drug treatment shrinks the cancer, but if CSCs still remain, they proliferate and gradually acquire anticancer drug resistance. Our study has shown that EpCAM-positive cells were very strongly expressed in the remaining cancer, even when treated by strong anticancer treatment such as HAIC. However, our study is limited because the amount of HCCs that were resected after the HAIC was very small. Nonetheless, EpCAM expression was increased in cases that were treated with TACE. In addition, our CSC model HCC cell line (EpCAM-positive cells in PLC/PRF/5) supported the result that EpCAM-positive cells had anticancer drug resistance. EpCAM-positive cells are not formed after the application of an anticancer drug to EpCAM-negative cells. Therefore, EpCAM-positive cells are thought to have anticancer drug ability.

In many studies, which were conducted *in vitro*, it was reported that CSCs have anticancer drug ability. Only a few studies have reported CSCs in the remaining cancer after treatment with anticancer drugs for HCC. Our study suggested that HAIC was very effective, but finally the remaining tumor required resection. If CSCs really exist, it is assumed that they will accumulate in the lesion. In our present study, we used clinical specimens to clarify the possible existence of CSCs in the remaining postanticancer drug-treated lesions. We showed that the EpCAM-positive cell of the PLC/PRF/5 had a characteristic of CSCs such as tumorigenicity or the differentiation ability in a previous study. Furthermore, we showed that EpCAM-positive cells had anticancer drug resistance in the model cell line of the CSCs in this study and EpCAM-positive cells accumulated in the treatment-resistant HCC from the resected specimens. We show that there are CSCs in an EpCAM-positive cells, and in other words, our study suggests the novel idea of many EpCAM-positive cells that are included in HBV-related HCC. Also the CSC showed possibility of being promoted by HBV. We assume that the development of CSCs could be clarified by further study of that target of the mechanism. Also, the development of new treatments may become possible in the future.

## 5. Conclusion

EpCAM is a good marker for CSC in HCC. Our results suggest that CSCs are highly expressed in hepatitis B and have the potential anticancer drug resistance.

## Figures and Tables

**Table 1:** Clinical profiles of 58 hepatocellular carcinoma patients. They had surgery without previous treatment.

	HBV	HCV	NBNC	
Number	18	23	17	
Age	56.0 ± 10.1	65.6 ± 8.3	69.8 ± 8.0	*p* = 0.0003
Sex (male/female)	14/4	15/8	15/2	*p* = 0.2369
Differentiation (wel/mod/por)	2/12/4	4/12/5	5/7/5	*p* = 0.7967
Tumor size (mm)	53.3 ± 52.3	42.3 ± 25.1	66.2 ± 42.3	*p* = 0.0756
ALT (IU/L)	61.1 ± 37.3	60.5 ± 48.7	32.6 ± 34.0	*p* = 0.0027
*γ*GTP (IU/L)	98.2 ± 86.8	79.3 ± 70.2	114.8 ± 153.3	*p* = 0.9223
Alb (g/dL)	4.1 ± 0.3	4.0 ± 0.4	4.0 ± 0.3	*p* = 0.5170
Plt (×10^3^/*μ*L)	161.2 ± 43.6	160.6 ± 48.2	213.6 ± 78.9	*p* = 0.0932
AFP (ng/mL)	1630.9 ± 2743.8	961.6 ± 2956.0	2717.8 ± 10983.9	*p* = 0.1753
AFP-L3 (%)	18.2 ± 22.7	16.0 ± 20.1	14.1 ± 23.9	*p* = 0.4964
PIVKA-II (AU/L)	48377.1 ± 132988.9	10912.2 ± 43623.6	16646.0 ± 39322.3	*p* = 0.5643

Data expressed as mean ± SD. ALT: alanine aminotransferase; *γ*GTP: *γ*-glutamyl transpeptidase; Alb: albumin; Plt: platelet; AFP: alpha-fetoprotein; AFP-L3 (%): LCA-reactive alpha-fetoprotein isoform; PIVKA-II: protein induced by vitamin K absence or antagonists-II. Sex and differentiation analyzed by *χ* ^2^ test. Other data analyzed by Kruskal-Wallis test.

**Table 2:** Expression grade of EpCAM in resected HCCs.

	Grade 0	Grade 1	Grade 2	Grade 3	Total
HBV	4	5	3	6	**18**
HCV	12	5	5	1	**23**
NBNC	10	0	4	3	**17**
Total	**26**	**10**	**12**	**10**	**58**

**Table 3:** Clinical profiles of surgery alone patients and patients who received prior treatment.

	Non prior treatment	Prior treatment (TACE or HAIC)	
Number	58	13	
Age	63.9 ± 10.3	62.6 ± 9.9	*p* = 0.7322
Sex (male/female)	14/4	15/8	*p* = 0.2369
Etiology (HBV/HCV/NBNC)	18/23/17	3/7/3	*p* = 0.6470
Tumor size (mm)	52.7 ± 40.8	28.6 ± 18.9	*p* = 0.0118
ALT (IU/L)	52.5 ± 42.7	35.8 ± 17.9	*p* = 0.3683
*γ*GTP (IU/L)	95.6 ± 104.7	58.5 ± 29.4	*p* = 0.4438
Plt (×10^3^/*μ*L)	176.3 ± 61.6	172.8 ± 112.2	*p* = 0.2553
Child-Pugh classification (*A*/*B*)	58/0	11/2	*p* = 0.024
AFP (ng/mL)	1684.1 ± 6325.6	2529.1 ± 7414.3	*p* = 0.6936

Data expressed as mean ± SD. Sex, etiology, and Child-Pugh were classification analyzed by *χ* ^2^ test. Other data were analyzed by Kruskal-Wallis test.

## References

[1] Lau W, Lai ECH (2008). Hepatocellular carcinoma: current management and recent advances. *Hepatobiliary and Pancreatic Diseases International*.

[2] Minami Y, Kudo M (2010). Radiofrequency ablation of hepatocellular carcinoma: current status. *World Journal of Radiology*.

[3] Ando E, Tanaka M, Yamashita F (2002). Hepatic arterial infusion chemotherapy for advanced hepatocellular carcinoma with portal vein tumor thrombosis: analysis of 48 cases. *Cancer*.

[4] Kondo M, Morimoto M, Numata K, Nozaki A, Tanaka K (2011). Hepatic arterial infusion therapy with a fine powder formulation of cisplatin for advanced hepatocellular carcinoma with portal vein tumor thrombosis. *Japanese Journal of Clinical Oncology*.

[5] Hotte SJ, Hirte HW (2002). BAY 43-9006: early clinical data in patients with advanced solid malignancies. *Current Pharmaceutical Design*.

[6] Bruix J, Raoul JL, Sherman M (2012). Efficacy and safety of sorafenib in patients with advanced hepatocellular carcinoma: subanalyses of a phase III trial. *Journal of Hepatology*.

[7] Bonnet D, Dick JE (1997). Human acute myeloid leukemia is organized as a hierarchy that originates from a primitive hematopoietic cell. *Nature Medicine*.

[8] Al-Hajj M, Wicha MS, Benito-Hernandez A, Morrison SJ, Clarke MF (2003). Prospective identification of tumorigenic breast cancer cells. *Proceedings of the National Academy of Sciences of the United States of America*.

[9] Singh SK, Clarke ID, Terasaki M (2003). Identification of a cancer stem cell in human brain tumors. *Cancer Research*.

[10] Visvader JE, Lindeman GJ (2008). Cancer stem cells in solid tumours: accumulating evidence and unresolved questions. *Nature Reviews Cancer*.

[11] Yin S, Li J, Hu C (2007). CD133 positive hepatocellular carcinoma cells possess high capacity for tumorigenicity. *International Journal of Cancer*.

[12] Ma S, Chan K, Hu L (2007). Identification and characterization of tumorigenic liver cancer stem/progenitor cells. *Gastroenterology*.

[13] Yang ZF, Ho DW, Ng MN (2008). Significance of CD90^+^ cancer stem cells in human liver cancer. *Cancer Cell*.

[14] Haraguchi N, Ishii H, Mimori K (2010). CD13 is a therapeutic target in human liver cancer stem cells. *The Journal of Clinical Investigation*.

[15] Yamashita T, Ji J, Budhu A (2009). EpCAM-positive hepatocellular carcinoma cells are tumor-initiating cells with stem/progenitor cell features. *Gastroenterology*.

[16] Terris B, Cavard C, Perret C (2010). EpCAM, a new marker for cancer stem cells in hepatocellular carcinoma. *Journal of Hepatology*.

[17] Kimura O, Takahashi T, Ishii N (2010). Characterization of the epithelial cell adhesion molecule (EpCAM)+ cell population in hepatocellular carcinoma cell lines. *Cancer Science*.

[18] Maetzel D, Denzel S, Mack B (2009). Nuclear signalling by tumour-associated antigen EpCAM. *Nature Cell Biology*.

[19] Yamashita T, Forgues M, Wang W (2008). EpCAM and *α*-fetoprotein expression defines novel prognostic subtypes of hepatocellular carcinoma. *Cancer Research*.

[20] Chemin I, Zoulim F (2009). Hepatitis B virus induced hepatocellular carcinoma. *Cancer Letters*.

[21] Arzumanyan A, Friedman T, Ng IOL, Clayton MM, Lian Z, Feitelson MA (2011). Does the hepatitis B antigen HBx promote the appearance of liver cancer stem cells?. *Cancer Research*.

[22] Wang C, Yang W, Yan H (2012). Hepatitis B virus X (HBx) induces tumorigenicity of hepatic progenitor cells in 3,5-diethoxycarbonyl-1,4-dihydrocollidine-treated HBx transgenic mice. *Hepatology*.

[23] Chisari FV, Ferrari C (1995). Hepatitis B virus immunopathogenesis. *Annual Review of Immunology*.

[24] Tanaka N, Moriya K, Kiyosawa K, Koike K, Gonzalez FJ, Aoyama T (2008). PPAR*α* activation is essential for HCV core protein-induced hepatic steatosis and hepatocellular carcinoma in mice. *The Journal of Clinical Investigation*.

[25] Kim H, Choi GH, Na DC (2011). Human hepatocellular carcinomas with “Stemness”-related marker expression: keratin 19 expression and a poor prognosis. *Hepatology*.

